# Supplementary material for: CAMSAPs and nucleation-promoting factors control microtubule release from γ-TuRC
Source: Nat Cell Biol. 2024 Feb 29;26(3):404–20. doi: 10.1038/s41556-024-01366-2 (PMC10940162; doi:10.1038/s41556-024-01366-2)
Supplement: Supplementary file 1 — Legends for Extended Data Figs. 1–5, Supplementary Tables 1–6, Videos 1–11 and Source data files 1–5. [file 41556_2024_1366_MOESM1_ESM.pdf]

nature cell biology

Article

https://doi.org/10.1038/s41556-024-01366-2

CAMSAPs and nucleation-promoting factors control microtubule release from γ-TuRC

In the format provided by the authors and unedited

Supplementary Video Legends

Supplementary Video 1. **Microtubule re-nucleation from γ-TuC in the presence of CDK5RAP2 and chTOG.** Videos of 10 min time-lapse images illustrating repeated microtubule nucleation from γ-TuC in the presence of 17.5 μM tubulin (17 μM unlabeled porcine tubulin and 0.5 μM HiLyte647-tubulin), 50 mM KCl and 30 nM premixed mCherry-CDK5RAP2 or 200 nM non-premixed chTOG-mCherry. Time-lapse images were acquired using TIRF microscope at 5 s time interval, constituting 121 frames and displayed at 12 fps.

Supplementary Video 2. **Microtubule release from γ-TuC in the presence of chTOG.** Video of 10 min time-lapse images illustrating the elongation of the minus end anchored to γ-TuC, purified using GCP3-GFP-SII, in the presence of 17.5 μM tubulin (~3% labeling), 50 mM KCl and 50 nM non-premixed chTOG-mCherry. Time-lapse images were acquired using TIRF microscope at 3 s time interval constituting 201 frames and displayed at 20 fps.

Supplementary Video 3. **CAMSAP3-mediated microtubule release from γ-TuRC purified using GCP3-GFP-SII.** Video of 10 min time-lapse images illustrating CAMSAP3 binding to γ-TuC-anchored minus ends and subsequent elongation of the CAMSAP3-stabilized minus ends in the presence of 25 μM tubulin (24.5 μM unlabeled porcine tubulin and 0.5 μM rhodamine-tubulin), 80 mM KCl and 5 nM SNAP-AF647-CAMSAP3. Time-lapse images were acquired using TIRF microscope at 20 s time intervals constituting 31 frames and displayed at 3 fps.

Supplementary Video 4. **Example of freely diffusing microtubule released from γ-TuC by CAMSAP3.** Video of 10 min time-lapse images illustrating CAMSAP3 binding to γ-TuC-anchored minus ends and very rapid release of microtubule upon subsequent elongation of the CAMSAP3-stabilized minus ends in the presence of 25 μM tubulin (~2% labeling), 80 mM KCl and 5 nM SNAP-AF647-CAMSAP3. Time-lapse images were acquired using TIRF microscope at 3 s time intervals constituting 201 frames and displayed at 20 fps.

Supplementary Video 5. **Example of CAMSAP3 binding to γ-TuC-anchored microtubule minus end without subsequent microtubule detachment.** Video of 10 min time-lapse images illustrating CAMSAP3 binding to γ-TuC-anchored minus end without subsequent microtubule release in the

presence of 25 μM tubulin (~2% labeling), 80 mM KCl and 5 nM SNAP-AF647-CAMSAP3. Time-lapse images were acquired using TIRF microscope at 3 s time interval constituting 201 frames and displayed at 20 fps.

Supplementary Video 6. **CAMSAP3-mediated microtubule detachment from γ-TuC followed by nucleation of a new microtubule from the same γ-TuC.** Video of 10 min time-lapse images illustrating CAMSAP3 binding to a γ-TuC-anchored microtubule minus end and subsequent release of microtubule followed by nucleation of a new microtubule from the same γ-TuC in the presence of 25 μM tubulin (~2% labeling), 80 mM KCl and 5 nM SNAP-AF647-CAMSAP3. Time-lapse images were acquired using TIRF microscope at 4 s time intervals constituting 151 frames and displayed at 15 fps.

Supplementary Video 7. **CAMSAP3-mediated microtubule release from γ-TuRC purified using GCP6-GFP-SII.** Video of 10 min time-lapse images illustrating CAMSAP3 binding to γ-TuC-anchored minus ends and subsequent elongation of the CAMSAP3-stabilized minus ends in the presence of 25 μM tubulin (~2% labeling), 80 mM KCl and 5 nM SNAP-AF647-CAMSAP3. Time-lapse images were acquired using TIRF microscope at 3 s time intervals constituting 201 frames and displayed at 20 fps.

Supplementary Video 8. **γ-TuC detachment from microtubule minus ends by CAMSAP2 and CAMSAP1.** Videos of 10 min time-lapse images illustrating CAMSAPs binding to γ-TuC-anchored minus ends and subsequent elongation of the CAMSAPs-stabilized minus ends in the presence of 25 μM tubulin (~2% labeling), 80 mM KCl and 40 nM mCherry-CAMSAP2 or 5 nM SNAP-AF647-CAMSAP1. Time-lapse images were acquired using TIRF microscope at 20 s time interval constituting 31 frames and displayed at 3 fps.

Supplementary Video 9. **Examples of CAMSAP2 and CAMSAP1 binding to γ-TuC-anchored microtubule minus ends without subsequent microtubule release.** Videos of 10 min time-lapse images time-lapse videos illustrating CAMSAPs binding to γ-TuC-anchored minus ends without subsequent microtubule release in the presence of 25 μM tubulin (~2% labeling), 80 mM KCl and 40 nM mCherry-CAMSAP2 or 5 nM SNAP-AF647-CAMSAP1. Time-lapse images were acquired

using TIRF microscope at 3 s (CAMSAP2, constituting 201 frames) and 20 s (CAMSAP1, constituting 31 frames) time intervals and displayed at 20 fps.

Supplementary Video 10. **γ-TuC displacement from microtubule minus ends by CAMSAP3 in the presence of nucleation-promoting factors.** Videos of 10 min time-lapse images illustrating CAMSAP3 binding to γ-TuC-anchored minus ends and subsequent elongation of the CAMSAP3-stabilized minus ends in the presence of 25 µM tubulin (~2% labeling), 80 mM KCl, 5 nM SNAP-AF647-CAMSAP3 and the indicated nucleation-promoting factors (30 nM mCherry-CDK5RAP2 or 30 nM mCherry-CLASP2 or 200 nM chTOG-mCherry). Time-lapse images were acquired using TIRF microscope at 20 s time interval constituting 31 frames and displayed at 3 fps.

Supplementary Video 11. **CAMSAP3 binding to γ-TuC-anchored microtubule minus ends in the presence of nucleation-promoting factors without subsequent microtubule release.** Videos of 10 min time-lapse images illustrating examples of CAMSAP3 binding to γ-TuC-anchored minus ends, but no subsequent microtubule release in the presence of 25 µM tubulin (~2% labeling), 80 mM KCl, 5 nM SNAP-AF647-CAMSAP3 and indicated nucleation-promoting factors (30 nM mCherry-CDK5RAP2 or 30 nM mCherry-CLASP2 or 200 nM chTOG-mCherry). Time-lapse images were acquired using TIRF microscope at 20 s (mCherry-CDK5RAP2, constituting 31 frames), 3 s (mCherry-CLASP2, constituting 201 frames) and 10 s (chTOG-mCherry, constituting 61 frames) time intervals and displayed at 20 fps.

Supplementary Table Legends

Supplementary Table 1. Mass-spectrometry analysis of purified GCP3-GFP-SII. List of proteins that were co-purified with endogenously tagged GCP3-GFP-SII.

Supplementary Table 2: iBAQ analysis of mass-spectrometry results of γ-TuRC samples purified using GCP3-GFP-SII or GCP6-GFP-SII. Estimation of protein abundance in γ-TuRC samples purified using GCP3-GFP-SII or GCP6-GFP-SII and analyzed by mass-spectrometry for iBAQ intensities. These iBAQ intensities of γ-TuRC core components along with other co-purified proteins were normalized to corresponding iBAQ intensity of GCP6 in that sample to calculate relative iBAQ ratios. This ratio provides an estimate of relative abundance and stoichiometries of core components of γ-TuRC in samples purified using GCP3-GFP-SII or GCP6-GFP-SII.

Supplementary Table 3. Mass-spectrometry analysis of purified CDK5RAP2. List of proteins identified by mass spectrometry that were co-purified with SII-mCherry-CDK5RAP2.

Supplementary Table 4. Mass-spectrometry analysis of purified CLASP2. List of proteins identified by mass spectrometry that were co-purified with SII-mCherry-CLASP2.

Supplementary Table 5. Mass-spectrometry analysis of purified chTOG. List of proteins identified by mass spectrometry that were co-purified with chTOG-mCherry-SII.

Supplementary Table 6. Mass-spectrometry analysis of purified GCP6-GFP-SII. List of proteins that were co-purified with endogenously tagged GCP6-GFP-SII.

Source data

Source data Extended Data Fig. 1. Unprocessed gels and Western blots.

Source data Extended Data Fig. 2. Unprocessed gels.

Source data Extended Data Fig. 3. Unprocessed gels and Western blots.

Source data Extended Data Fig. 4. Unprocessed Western blots.

Statistical source data. Numerical and statistical source data.
