# Supplementary material for: CAMSAPs and nucleation-promoting factors control microtubule release from γ-TuRC
Source: Nat Cell Biol. 2024 Feb 29;26(3):404–20. doi: 10.1038/s41556-024-01366-2 (PMC10940162; doi:10.1038/s41556-024-01366-2)
Supplement: Supplementary file 2 — Reporting Summary [file 41556_2024_1366_MOESM2_ESM.pdf]

nature portfolio

Corresponding author(s): Kai Jiang and Anna Akhmanova

Last updated by author(s): Jan 19, 2024

# Reporting Summary

Nature Portfolio wishes to improve the reproducibility of the work that we publish. This form provides structure for consistency and transparency in reporting. For further information on Nature Portfolio policies, see our Editorial Policies and the Editorial Policy Checklist.

## Statistics

For all statistical analyses, confirm that the following items are present in the figure legend, table legend, main text, or Methods section.

| n/a | Confirmed | |
|---|---|---|
| ☐ | ☒ | The exact sample size (*n*) for each experimental group/condition, given as a discrete number and unit of measurement |
| ☐ | ☒ | A statement on whether measurements were taken from distinct samples or whether the same sample was measured repeatedly |
| ☐ | ☒ | The statistical test(s) used AND whether they are one- or two-sided<br>*Only common tests should be described solely by name; describe more complex techniques in the Methods section.* |
| ☒ | ☐ | A description of all covariates tested |
| ☐ | ☒ | A description of any assumptions or corrections, such as tests of normality and adjustment for multiple comparisons |
| ☐ | ☒ | A full description of the statistical parameters including central tendency (e.g. means) or other basic estimates (e.g. regression coefficient) AND variation (e.g. standard deviation) or associated estimates of uncertainty (e.g. confidence intervals) |
| ☐ | ☒ | For null hypothesis testing, the test statistic (e.g. *F*, *t*, *r*) with confidence intervals, effect sizes, degrees of freedom and *P* value noted<br>*Give P values as exact values whenever suitable.* |
| ☒ | ☐ | For Bayesian analysis, information on the choice of priors and Markov chain Monte Carlo settings |
| ☒ | ☐ | For hierarchical and complex designs, identification of the appropriate level for tests and full reporting of outcomes |
| ☒ | ☐ | Estimates of effect sizes (e.g. Cohen's *d*, Pearson's *r*), indicating how they were calculated |

*Our web collection on statistics for biologists contains articles on many of the points above.*

## Software and code

Policy information about availability of computer code

| | |
|---|---|
| Data collection | MetaMorph 7.10.2.240 software, Nikon NIS Br software, MAPS automated acquisition software, LI-COR Image Studio software 5.2.5, Refeyn SamuxMP software, Leica LAS X software. Software details are also provided in the methods section. |
| Data analysis | ImageJ 1.45s, ImageJ 1.53c (Fiji), ImageJ plugin KymoResliceWide v.0.4 (https://github.com/ekatrukha/KymoResliceWide), custom made JAVA plugin, ImageJ plugin ComDet v.0.5.4 (https://github.com/ekatrukha/ComDet), ImageJ plugin DoM_Utrecht v.1.1.6 (https://github.com/ekatrukha/DoM_Utrecht), custom MATLAB script for GFP stoichiometry analysis (https://doi.org/10.6084/m9.figshare.23943150.v4), Proteome Discoverer version 1.4.0.288 and 2.4, Mascot version 2.4.1, Sequest HT, MaxQuant version 2.1.3.0, Refeyn DiscoverMP, Python scripts, CTFFIND4, RELION version 3.1, UCSF Chimera and ChimeraX, PyMOL, custom MATLAB script, Microsoft Excel for Microsoft 365, GraphPad Prism 9. |

For manuscripts utilizing custom algorithms or software that are central to the research but not yet described in published literature, software must be made available to editors and reviewers. We strongly encourage code deposition in a community repository (e.g. GitHub). See the Nature Portfolio guidelines for submitting code & software for further information.

## Data

Policy information about availability of data

All manuscripts must include a data availability statement. This statement should provide the following information, where applicable:

- Accession codes, unique identifiers, or web links for publicly available datasets
- A description of any restrictions on data availability
- For clinical datasets or third party data, please ensure that the statement adheres to our policy

Previously published data that were re-analysed here are available, for γ-tubulin bound to GCP2 under accession code PDB ID: 6V6S, for β-tubulin subunits within a microtubule, PDB ID: 2HXF and EMD-5193, for γ-TuSC, PDB ID: 5FLZ. Custom MATLAB script developed for GFP stoichiometry analysis is available on https://doi.org/10.6084/m9.figshare.23943150.v4. Mass spectrometry data have been deposited to the ProteomeXchange Consortium via the PRIDE partner repository with the dataset identifier PXD048637. All data that support the conclusions are either available in the manuscript itself or available from the authors on request.

## Human research participants

Policy information about studies involving human research participants and Sex and Gender in Research.

| | |
|---|---|
| Reporting on sex and gender | N/A |
| Population characteristics | N/A |
| Recruitment | N/A |
| Ethics oversight | N/A |

Note that full information on the approval of the study protocol must also be provided in the manuscript.

# Field-specific reporting

Please select the one below that is the best fit for your research. If you are not sure, read the appropriate sections before making your selection.

☒ Life sciences ☐ Behavioural & social sciences ☐ Ecological, evolutionary & environmental sciences

For a reference copy of the document with all sections, see nature.com/documents/nr-reporting-summary-flat.pdf

# Life sciences study design

All studies must disclose on these points even when the disclosure is negative.

| | |
|---|---|
| Sample size | No statistical methods were used to predict the sample size. All datasets were pooled from at least three or more independent experiments. Sample size was chosen based on the reproducibility, our previous experience or the standards in the field. Data distribution was assumed to be normal but this was not formally tested. In each case, sample size, number of independent experiments and statistical tests, when used, along with p values were indicated in the figure panels, legends or in the statistical and reproducibility subsection within the methods section. |
| Data exclusions | No data were excluded from the analyses. |
| Replication | Each experimental condition was repeated at least three times or more unless stated otherwise. All attempts at replication were successful. |
| Randomization | No randomization was performed in our study as samples were not required to be allocated into experimental groups. |
| Blinding | Investigators were not blinded to group allocation during data collection and analyses as group allocation was not required. Data was collected immediately after each experiment was performed for each experimental condition with different proteins or different protein concentrations, and therefore blinding was not possible. The analyses were done using automated methods or using strictly defined parameters as mentioned in the methods section, therefore preventing any subjective error. |

# Reporting for specific materials, systems and methods

We require information from authors about some types of materials, experimental systems and methods used in many studies. Here, indicate whether each material, system or method listed is relevant to your study. If you are not sure if a list item applies to your research, read the appropriate section before selecting a response.



March 2021

## Materials & experimental systems

| n/a | Involved in the study |
| --- | --- |
| ☐ | ☒ Antibodies |
| ☐ | ☒ Eukaryotic cell lines |
| ☒ | ☐ Palaeontology and archaeology |
| ☒ | ☐ Animals and other organisms |
| ☒ | ☐ Clinical data |
| ☒ | ☐ Dual use research of concern |

## Methods

| n/a | Involved in the study |
| --- | --- |
| ☒ | ☐ ChIP-seq |
| ☒ | ☐ Flow cytometry |
| ☒ | ☐ MRI-based neuroimaging |

# Antibodies

**Antibodies used**

Commercial antibodies used in this study are listed here:
rabbit anti-CAMSAP2, Proteintech, Cat#17880-1-AP, RRID:AB_2068826; rat anti-α-tubulin, clone YL1/2, Pierce, Cat#MA1-80017, RRID:AB_2210201; goat anti-Rabbit and anti-Rat IgG Alexa Fluor -488, -594, -647, Molecular Probes, Cat#A-11034, Cat#A-11012, Cat#A-11006, Cat#A-11007; rabbit polyclonal anti-GFP, Abcam, ab290; mouse anti-GCP3, Santa Cruz, sc-373758; mouse monoclonal anti-GCP6, Santa Cruz, sc-374063; mouse monoclonal anti-GCP5, Santa Cruz, sc-365837; mouse monoclonal anti-GCP2, Santa Cruz, sc-377117; rabbit polyclonal anti-GCP4, ThermoFisher, PA5-30557; mouse monoclonal anti-γ-tubulin, Sigma, Cat#T6557, GTU-88; rabbit polyclonal anti-CDK5RAP2, Bethyl Laboratories, A300-554A; mouse monoclonal anti-Ku80, BD Biosciences, 611360; goat anti-rabbit IRDye-800CW, Cat#926-32211 and goat anti-mouse IRDye-680LT, Cat#926-68020 from Li-Cor Biosciences, Lincoln, LE. Detailed information on commercial antibodies can also be found in the methods section. The biotinylated anti-GFP nanobody and rat monoclonal anti-EB1 antibody were home-made. The same rat monoclonal anti-EB1 antibody (KT51) is also available through Abcam (#ab53358).

**Validation**

Commercial antibodies were validated in species mentioned above for immunofluorescence and western blots as noted on manufacturer's website.
Home-made biotinylated anti-GFP nanobody:
Katrukha, E. A., Mikhaylova, M., van Brakel, H. X., van Bergen En Henegouwen, P. M., Akhmanova, A., Hoogenraad, C. C., & Kapitein, L. C. (2017). Probing cytoskeletal modulation of passive and active intracellular dynamics using nanobody-functionalized quantum dots. Nature communications, 8, 14772. https://doi.org/10.1038/ncomms14772
Home-made anti-EB1 antibody:
Komarova, Y., Lansbergen, G., Galjart, N., Grosveld, F., Borisy, G.G. & Akhmanova, A. EB1 and EB3 control CLIP dissociation from the ends of growing microtubules. Mol Biol Cell 16, 5334-5345 (2005). https://doi.org/10.1091/mbc.e05-07-0614

# Eukaryotic cell lines

Policy information about cell lines and Sex and Gender in Research

**Cell line source(s)**

Human embryonic kidney 239T (HEK293T) cells (Cat#CRL-3216) were obtained from ATCC and used for generating knock-in cell lines.
hTERT RPE-1 (RPE1) wild type cells (Cat#CRL-4000) are available on ATCC.
RPE1 AKAP450 knockout and RPE1 AKAP450/CDK5RAP2/MMG triple knockout cell lines were previously published:
Wu, J., de Heus, C., Liu, Q., Bouchet, B. P., Noordstra, I., Jiang, K., Hua, S., Martin, M., Yang, C., Grigoriev, I., Katrukha, E. A., Altelaar, A. F. M., Hoogenraad, C. C., Qi, R. Z., Klumperman, J., & Akhmanova, A. (2016). Molecular Pathway of Microtubule Organization at the Golgi Apparatus. Developmental cell, 39(1), 44–60. https://doi.org/10.1016/j.devcel.2016.08.009
RPE1 AKAP450/CDK5RAP2/MMG triple knockout cell line mentioned above was used for generating transgenic cell line stably expressing GFP-CDK5RAP2.
RPE1 wild type and RPE1 AKAP450 knockout transgenic cell lines stably expressing GFP-CDK5RAP2 were previously published:
Chen, F., Wu, J., Iwanski, M. K., Jurriens, D., Sandron, A., Pasolli, M., Puma, G., Kromhout, J. Z., Yang, C., Nijenhuis, W., Kapitein, L. C., Berger, F., & Akhmanova, A. (2022). Self-assembly of pericentriolar material in interphase cells lacking centrioles. eLife, 11, e77892. https://doi.org/10.7554/eLife.77892

**Authentication**

ATCC performs short-tandem repeat profiling for cell line authentication, and no additional cell line authentication was performed.

**Mycoplasma contamination**

The cell lines were routinely checked for mycoplasma contamination using LT07-518 Mycoalert assay and has been verified as mycoplasma free.

**Commonly misidentified lines**
(See ICLAC register)

The cell lines used are not present in the list of commonly misidentified lines.
