# Supplementary material for: CAMSAPs and nucleation-promoting factors control microtubule release from γ-TuRC
Source: Nat Cell Biol. 2024 Feb 29;26(3):404–20. doi: 10.1038/s41556-024-01366-2 (PMC10940162; doi:10.1038/s41556-024-01366-2)

# Source data extended data figure 1

**Extended data Fig. 1b (uncropped agarose gel)**

**a** HEK293T genomic DNA

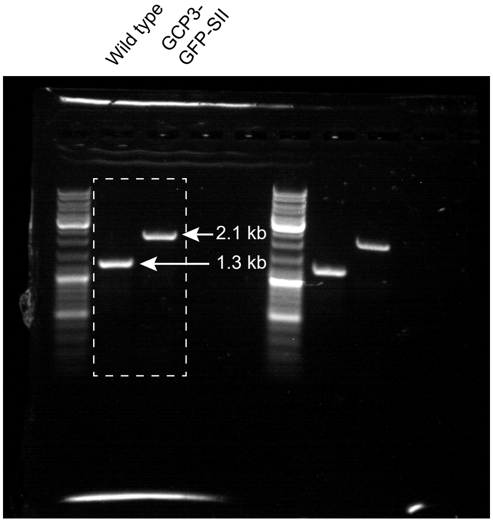


**Extended data Fig. 1d (uncropped western blots)**

**b** HEK293T cell lysates

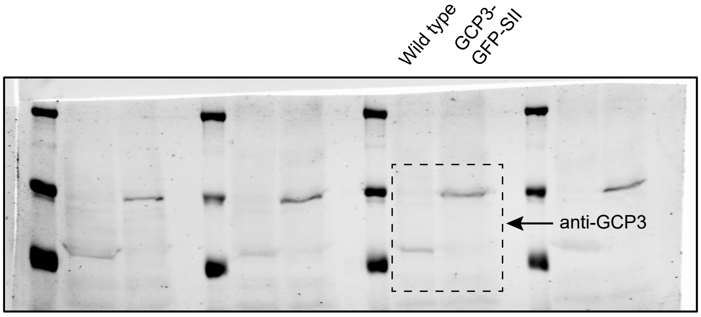


**Extended data Fig. 1f (uncropped western blots)**

**c**

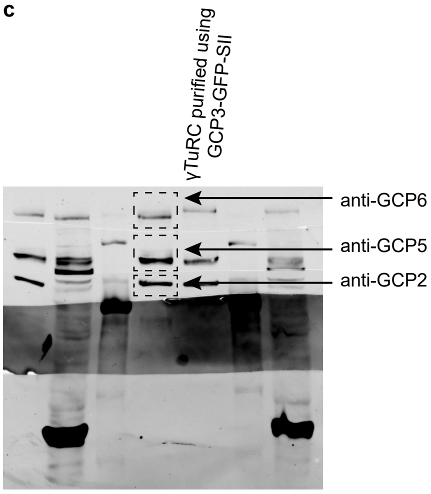


**d**

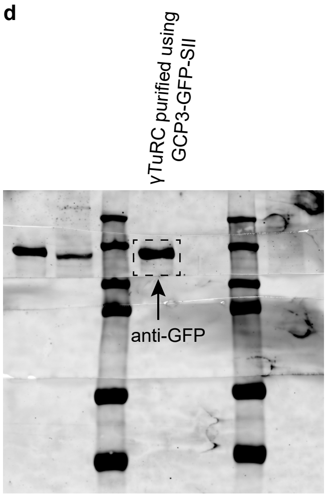


**e**

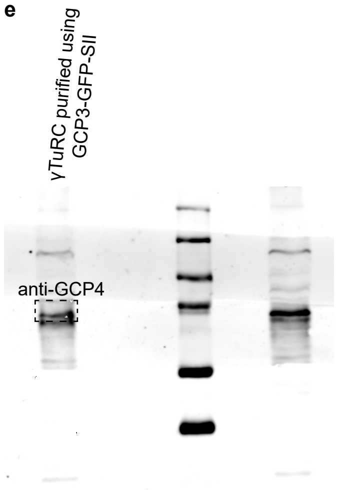


**f**

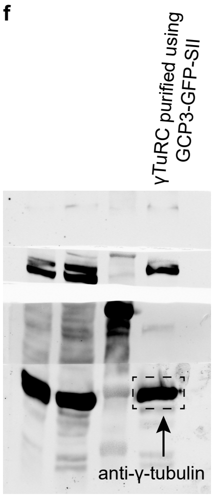


**Extended data Fig. 1h (uncropped western blots)**

**g**

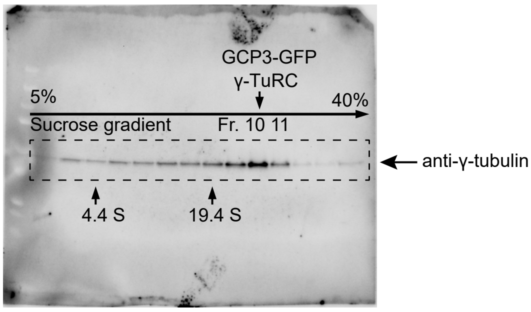


**Extended data Fig. 1h (uncropped Coomassie-stained gels)**

**h**

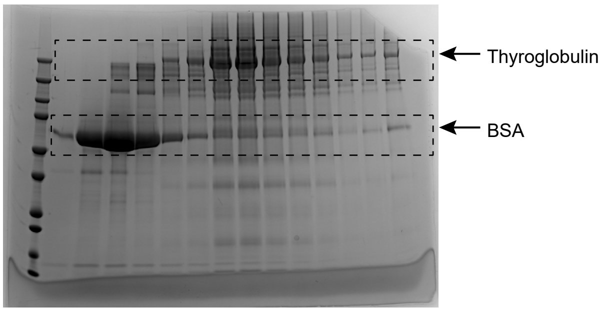

Supplement: Supplementary file 16 — Unprocessed gels and western blots. [file 41556_2024_1366_MOESM16_ESM.pdf]
