# Supplementary figures and images for: CAMSAPs and nucleation-promoting factors control microtubule release from γ-TuRC
Source: Nat Cell Biol. 2024 Feb 29;26(3):404–20. doi: 10.1038/s41556-024-01366-2 (PMC10940162; doi:10.1038/s41556-024-01366-2)

## Source data extended data figure 2

Extended data Fig. 2b (uncropped Coomassie-stained gels)

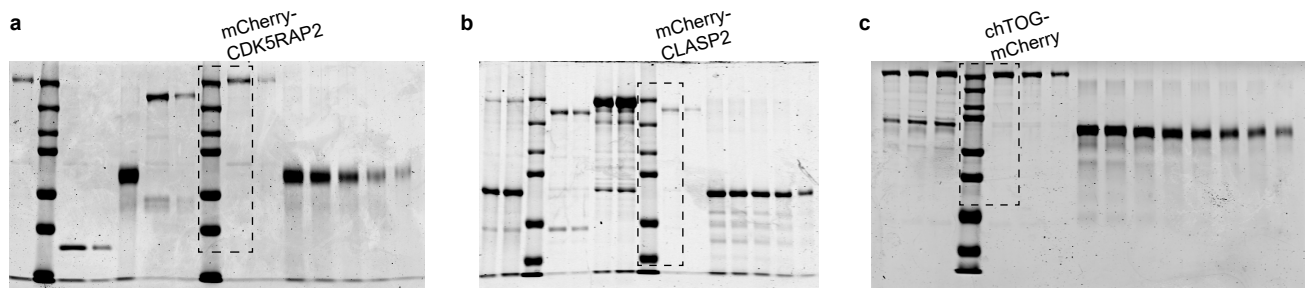

Supplement: Supplementary file 17 — Unprocessed gels. [file 41556_2024_1366_MOESM17_ESM.pdf]

Source data extended data figure 4

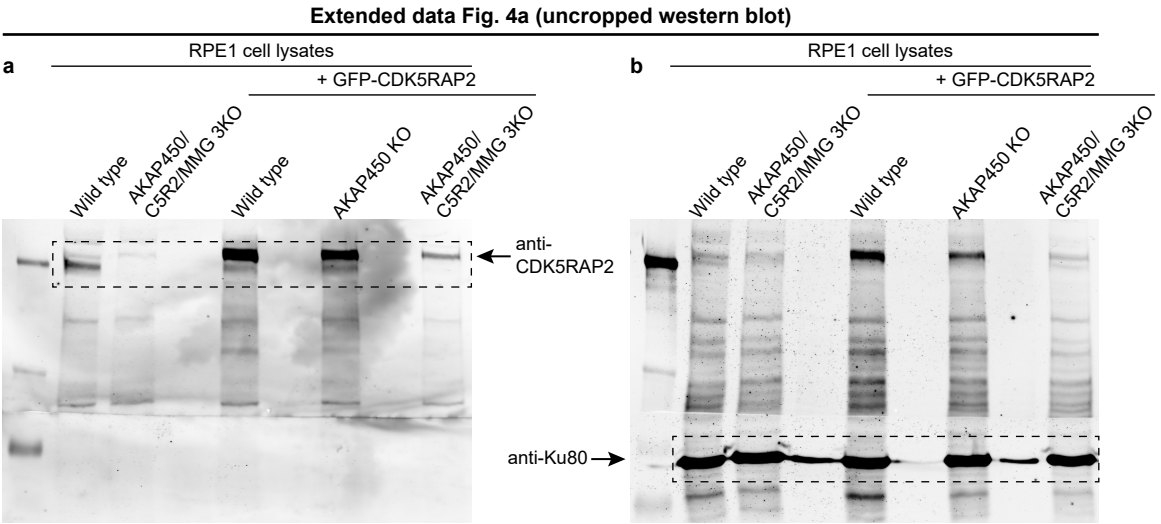

Supplement: Supplementary file 19 — Unprocessed western blots. [file 41556_2024_1366_MOESM19_ESM.pdf]
