# Supplementary material for: CAMSAPs and nucleation-promoting factors control microtubule release from γ-TuRC
Source: Nat Cell Biol. 2024 Feb 29;26(3):404–20. doi: 10.1038/s41556-024-01366-2 (PMC10940162; doi:10.1038/s41556-024-01366-2)

Source data extended data figure 3

Extended data Fig. 3d
(uncropped Coomassie-stained gel)

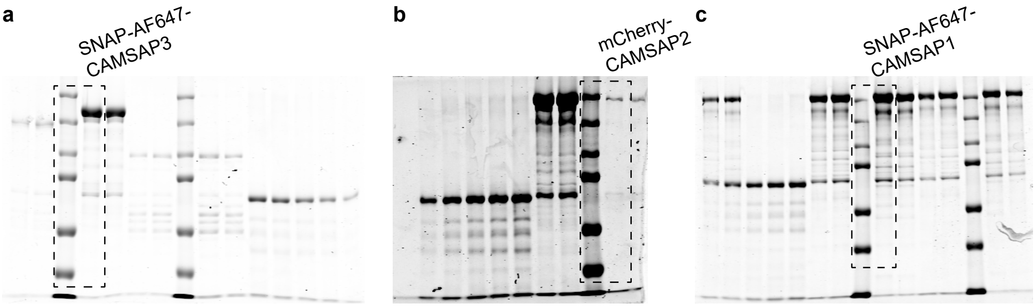

Extended data Fig. 3h
(uncropped western blot)

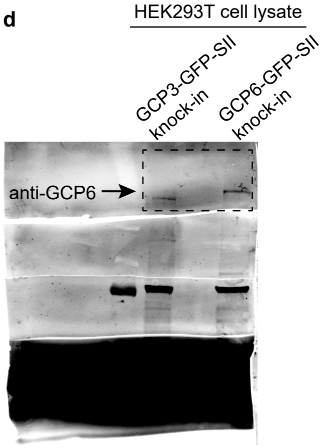

Supplement: Supplementary file 18 — Unprocessed gels and western blots. [file 41556_2024_1366_MOESM18_ESM.pdf]
